# Effects of *Fusarium* Mycotoxin Exposure on Lipid Peroxidation and Glutathione Redox System in the Liver of Laying Hens

**DOI:** 10.3390/antiox10081313

**Published:** 2021-08-20

**Authors:** Szabina Kulcsár, Benjámin Kövesi, Krisztián Balogh, Erika Zándoki, Zsolt Ancsin, Balláné Erdélyi Márta, Miklós Mézes

**Affiliations:** 1 Department of Feed Safety, Gödöllő Campus, Institute of Physiology and Nutrition, Hungarian University of Agriculture and Life Sciences, H-2100 Gödöllő, Hungary; Szabina.kulcsar@gmail.com (S.K.); Benjamin.kovesi@gmail.com (B.K.); Balogh.Krisztian.Milan@uni-mate.hu (K.B.); Ancsin.Zsolt@uni-mate.hu (Z.A.); Ballane.Erdelyi.Marta@uni-mate.hu (B.E.M.); 2 MTA-KE-SZIE Mycotoxins in the Food Chain Research Group, Institute of Physiology and Nutrition, Hungarian University of Agriculture and Life Sciences, H-7400 Kaposvár, Hungary; Zandoki.Erika@mkk.szie.hu

**Keywords:** T-2 toxin, deoxynivalenol, fumonisin B_1_, oxidative stress, glutathione redox system, gene expression, laying hen

## Abstract

It has been proven by several studies that *Fusarium* mycotoxins induce oxidative stress in animals, consequently inducing lipid peroxidation, which the glutathione system can neutralize. A short-term (3-day) in vivo feeding trial was performed with laying hens using a double dose of the EU recommendation for mycotoxin contamination (T-2 toxin 0.5 mg/kg feed; deoxynivalenol (DON) 10 mg/kg feed; fumonisin B_1_ (FB1) 40 mg/kg feed). Some lipid peroxidation and glutathione redox system parameters and gene expression levels were measured in the liver. The results show that FB1 significantly decreased the reduced glutathione (GSH) content and the activity of glutathione peroxidase (GPx) compared to the control and the two other mycotoxin-treated groups on day 3. Lipid peroxidation was affected by all three mycotoxins. Significantly lower values were observed in the case of conjugated dienes for all of the three mycotoxins and malondialdehyde concentration as an effect of DON on day 3. T-2 toxin and DON upregulated the expression of the *GPX4* gene. The results show that *Fusarium* mycotoxins had different effects at the end of the trial. The FB1 exposure caused a decrease in the glutathione redox markers, while DON decreased the formation of malondialdehyde. The results suggest that the *Fusarium* mycotoxins investigated individually differently activated the antioxidant defense and caused low-level oxidative stress at the dose applied.

## 1. Introduction

Intensive agriculture and climate change can lead to the proliferation of microscopic fungi, which can cause severe economic and health damage through their toxin production. *Fusarium* species infect cereals, such as wheat, barley, oats, and maize, worldwide. According to the Biomin Worldwide Mycotoxin Survey [1], the percentage of positive samples of finished feeds in Europe was 65% for deoxynivalenol (DON) with an average of 268 µg/kg (maximum: 18,300 µg/kg), 36% for T-2 toxin with an average of 15 µg/kg (maximum: 296 µg/kg), and 64% for fumonisin B_1_ (FB1) with an average of 358 µg/kg (maximum: 11,210 µg/kg). The contamination of cereals with *Fusarium* toxins may cause feed-borne toxicosis in farm animals [2]. One of the most important groups of mycotoxins is the fusariotoxins. Based on their economic importance and practical occurrence, the most important members of this toxin family are DON, T-2 toxin, fumonisin B_1_, zearalenone, and their toxic metabolites [3]. As poultry feed is primarily based on maize, research on fusariotoxins needs to be extended to poultry breeds. There are different methods for decreasing the detrimental effects of mycotoxins. Among them, a well-known method is the use of adsorbents with the capacity to bind mycotoxins in the gastrointestinal tract [4]. This method successfully eliminates the risk of certain mycotoxins but is not effective for all of the mycotoxins relevant to the poultry industry. Biotransformation has been proven useful for the detoxification of mycotoxins by modification of mycotoxins into nontoxic metabolites [5].

Poultry species have different levels of resistance to mycotoxins. For instance, T-2 toxin is highly toxic [6], while DON is less toxic than T-2 toxin [7], and the poultry species are relatively tolerant to the toxic effects of FB1 [8].

In nature, several mycotoxins co-occur, so several mycotoxin interactions have recently been studied in in vivo and in vitro experiments. However, to estimate the nature of the interactions, we need to know the individual effects. Farm animals typically intake mycotoxins orally with feed, and mycotoxins induce abrasions and local inflammation through skin contact [9]. In broiler chickens, trichothecene mycotoxins have mainly nonspecific effects, such as genotoxic, cytotoxic, and immunomodulatory effects. Besides these, they harm the digestive system, liver, and nervous system, thereby reducing production traits [10]. T-2 toxin and DON metabolism may be initiated in the intestinal tract by certain microbial enzymes [11]. DON is converted to deepoxy DON (DOM-1), and T-2 toxin is converted to other metabolites, such as HT-2 toxin [11]. Trichothecene mycotoxins, such as T-2/HT-2 toxin and DON, have epoxide groups, making them particularly reactive compounds. This can be one possible explanation for their dermatotoxic effects [12]. T-2/HT-2 toxin and DON inhibit protein and DNA synthesis in eukaryotic cells [13] at the level of ribosomes [14]. Due to their inhibitory effect on protein synthesis, hematopoietic and lymphoid tissues are particularly sensitive to these mycotoxins [15]. T-2/HT-2 toxin and DON are well-known immunosuppressive compounds [16]. DON and T-2 toxin exposure decreased the antibody titer after vaccination against infectious bronchitis virus and Newcastle disease, reducing vaccination efficacy [17,18]. They also have a neurotoxic effect by influencing the signaling processes of cells [19] and are also emetic in this context [20].

Of the fumonisins discovered (fumonisin B_1_, B_2_, B_3_, etc.), FB1 is the most effective form from an animal and human health point of view. FB1 is structurally similar to sphingosine; therefore, it explicitly inhibits the enzyme ceramide synthetase, thus interfering with sphingolipid biosynthesis [21] and altering the metabolism of phospholipids and polyunsaturated fatty acids [22]. By inhibiting sphingolipid metabolism, fumonisins can cause cell damage and apoptotic cell death by damaging biological membranes. In addition, depletion of sphingolipids impairs the barrier function of cell membranes and increases the permeability of endothelial cells.

Oxidative stress and lipid peroxidation are well-known effects of trichothecene mycotoxins and fumonisin. T-2/HT-2 toxin and FB1, through the induction of lipid peroxidation processes, may reduce the function of liver microsomes [23], which in turn may inhibit the function of the xenobiotic transforming (cytochrome P450) system [24]. In xenobiotic transformation, besides the cytochrome P450 enzymes, the amount and activity of the biological antioxidant defense, specifically the glutathione redox system, can vary significantly depending on the degree of oxidative stress. Based on previous research [25,26], it can be said that in connection with biochemical changes in cells, trichothecene mycotoxins increase the intensity of lipid peroxidation processes, which affects the functioning of the biological antioxidant system. Results of several previous short-term studies showed that trichothecene mycotoxins induce lipid peroxidation and activate the glutathione redox system in broiler chicken but at high dose levels [27]. However, another previous study did not prove the effect of T-2 toxin on induction of lipid peroxidation even at the same dose range in broiler chicken [28]. The same contradictory results were found in DON and broiler chicken in long-term studies with high doses [29,30]. Initiation of lipid peroxidation by fumonisin B_1_ was found in a high dose in a long-term study with broiler chicken [31].

Based on the results mentioned above, the present study aimed to explore the effect of experimentally contaminated feeds with these mycotoxins individually during short-term oral exposure on the initiation and termination phases of lipid peroxidation in the liver, which is important in the metabolism and storage of mycotoxins and parallels with glutathione redox system activity. In addition, the relative expression of genes encoding certain enzymes of the glutathione redox system was also determined. However, there are still many questions that remain open about the effects of T-2 toxin, DON, and FB1, especially on the processes involved in the oxygen free radical formation and the mechanism of their effects on the glutathione redox system. Furthermore, the short-term effects of T-2 toxin, DON, and FB1 on birds are currently even less known, so we chose economically important laying hens as a model organism.

We already have a wealth of knowledge about the toxic effects of fusariotoxins based on changes in the parameters of individual biological processes and the mechanisms that regulate them. Still, much of this is based on in vitro model studies or in vivo, but usually sublethal, long-term studies. In the present study, the effects of fusariotoxins were investigated individually, which means that we can assess the mechanism of each mycotoxin. Still, it may provide a basis for multi-mycotoxin studies.

## 2. Materials and Methods

### 2.1. Mycotoxin Production

T-2 toxin was produced by Fusarium sporotrichioides (NRRL 3299), DON was produced by Fusarium graminearum (NRRL 5883), and FB1 was produced by Fusarium verticillioides (MRC 826) strain on corn substrate according to the method of Fodor et al. [32]. These strains primarily produce only those mycotoxins, but the presence of other mycotoxins should be considered moderately. Therefore, according to its measured mycotoxin content, an appropriate amount of the fungal culture was added to the poultry feed. The DON, 3-acetyl DON (3-AcDON), and 15-acetyl DON (15-AcDON) contents of the artificially contaminated and control feeds [33]; T-2/HT-2 toxin [34]; and FB1 [35] were measured by HPLC equipped with fluorescence detector after immune-affinity clean up. The control feed did not contain detectable amounts of T-2/HT-2 toxin, DON, or FB1. The predicted and measured mycotoxin contents of the feeds are shown in Table 1.

### 2.2. Animals and Experimental Design

The experimental animals were Tetra SL laying hens (49 weeks of age, 90% average daily egg production, *n* = 78). Four groups were formed, one control and three treated groups, each containing 18 animals, and 6 animals were absolute control. Drinking water was provided ad libitum, but a restricted daily feeding protocol was used (150 g feed/day/bird). The calculated nutrient content of the laying hen diet is given in Table 2.

A short-term, 3-day, in vivo feeding trial, was performed using 2× the recommended mycotoxin content of poultry feed level in the EU [36]. The predicted doses were 0.5 mg/kg T-2/HT-2 toxin, 10 mg/kg DON, and 40 mg/kg FB1. However, according to a recent worldwide survey, these contamination levels may be present in animal feeds [1]. The control group was fed the same diet without experimental mycotoxin contamination, and its mycotoxin content was lower than the limit of quantification in the case of all three mycotoxins.

Laying hens were kept in deep litter condition with natural light regimen (12 L/12 D). The experiment started after 12 h of feed deprivation. Six animals were sampled randomly at 0 h as absolute control, and six animals from each experimental group were sampled on days 1, 2, and 3. After cervical dislocation, liver samples were taken and were put into liquid nitrogen. Liver samples were stored at −70 °C until biochemical and gene expression studies.

The experiment was performed in accordance with the European Communities Council Directive (86/609 EEC). The experimental protocol was approved by the Food Chain Safety, Land Use, Plant and Soil Protection and Forestry Directorate of the Pest County Governmental Office (PE/EA/1964-7/2017).

### 2.3. Biochemical Analyses

The amount of reduced glutathione (GSH) and activity of glutathione peroxidase (GPx), as well as the markers of the initiation phase of lipid peroxidation, conjugated dienes (CDs) and conjugated trienes (CTs), and the terminal phase marker, the concentration of malondialdehyde (MDA), were measured in the liver homogenate (9-fold volume isotonic saline). Levels of CDs and CTs were determined after extraction of the lipid content of the liver with 2,2,4-trimethylpentane, and the absorbance of the CDs was measured at 232 nm, while that of the CTs was measured at 268 nm [37]. The MDA content was determined in an acidic medium by complex formation with 2-thiobarbituric acid [38]. Among members of the glutathione redox system, GSH content was measured according to Sedlak and Lindsay [39], and the activity of GPx was measured according to Lawrence and Burk [40]. GSH content and GPx activity were calculated to protein content of the 10,000 g supernatant fraction of liver homogenates, which was measured with Folin’s phenol reagent [41].

### 2.4. Gene Expression Studies with Quantitative Real-Time PCR

Total RNA was purified from 6–10 mg liver with NucleoZOL reagent (Macherey-Nagel, Düren, Germany), according to the manufacturer’s manual. Genomic DNA was removed from the purified RNA samples by DNase I (Thermo Fisher Scientific, San Jose, CA, USA) as proposed by the manufacturer. RNA concentration, purity, and integrity were examined in a 2% agarose gel using a NanoPhotometer (Implant, Munich, Germany), where samples for the OD 260/280 index above 2.0 were accepted. According to the recommended protocol, cDNA was generated from 1 µg of RNA per sample by random nonamer reverse transcription (RevertAID Reverse Transcriptase, Thermo Fisher Scientific, San Jose, CA, USA). Pools were created from the cDNA for each treatment group, from the same amount of cDNA per individual (*n* = 6), and those pooled samples were used for qPCR measurements. The expression of phospholipid hydroperoxide glutathione peroxidase (*GPX4*), glutathione synthetase (*GSS*), and glutathione reductase (*GSR*) target genes, as well as glyceraldehyde-3-phosphate dehydrogenase (*GAPDH*) as endogenous control gene, was examined by quantitative real-time PCR using the duplex qPCR method. The *GAPDH* gene as an endogenous control was selected based on literature data [42] because it has no interaction with oxidative stress or mycotoxins. Specific primers designed for the target and the household genes (Table 3) and dual-labeled (minor groove binder (MGB)) TaqMan probes labeled with different fluorescent dyes (Table 4) allowed the simultaneous analysis of two gene products. Primers and probes were designed using Primer Express 3.0.1 (Thermo Fisher Scientific, San Jose, CA, USA) software.

Measurements were performed with StepOnePlus real-time PCR system (Thermo Fisher Scientific, San Jose, CA, USA) using Maxima Probe qPCR Master Mix (1 × final concentration) (Thermo Fisher Scientific, San Jose, CA, USA) as described previously [43]. The VIC and FAM signals were read at 72 °C in each cycle at the end of the extension period. In addition, the specificity of the PCR product and the presence of primary dimers were checked by gel electrophoresis.

Ct values for both target and control genes were determined with StepOnePlus (v2.2) software (Thermo Fisher Scientific, San Jose, CA, USA), and delta Ct (ΔCt) and delta-delta Ct (ΔΔCt) values were calculated. Finally, the RQ (relative quantification; RQ = 2^−ΔΔCt^) values were calculated [44].

### 2.5. Statistical Analysis

Data are expressed as mean ± standard deviation (SD). GraphPad Prism 6.07 software (GraphPad Software, San Diego, CA, USA) was used for the statistical evaluation. Normality was confirmed with the Kolmogorov–Smirnov, test and the homogeneity of variance was confirmed with the Bartlett test. Data passing both tests were analyzed by one-way ANOVA and Tukey’s post hoc test (*p* < 0.05). Kruskal–Wallis test was used for pairwise comparisons (*p* < 0.05).

## 3. Results

### 3.1. Effect of T-2 Toxin, DON, and FB1 on Lipid Peroxidation Parameters in the Liver of Laying Hens

The levels of initiation phase markers, CDs and CTs, and terminal phase marker of lipid peroxidation, MDA concentration, are shown in Table 5. The CD levels were significantly (*p* < 0.05) lower as an effect of the short-term exposure with all three examined mycotoxins compared to the control. In addition, the MDA concentration was significantly (*p* < 0.05) lower in the liver of laying hens fed a DON-contaminated diet as compared to the control and T-2 toxin group at the end of the experiment (day 3). There were no significant differences in the amount of CT levels.

### 3.2. Effect of T-2 Toxin, DON, and FB1 on Markers of the Glutathione Redox System in the Liver of Laying Hens

The amount of GSH and the activity of GPx were significantly (*p* < 0.05) lower compared to the control and the two trichothecene-treated groups on day 3 as an effect of FB1 (Table 6).

### 3.3. Effect of T-2 Toxin, DON, and FB1 on the Relative Expression of the GPX4, GSS, and GSR Genes in the Liver of Laying Hens

The relative gene expression of *GPX4* was significantly elevated on day 1 and day 3 of T-2 toxin exposure (*p* < 0.05) and as an effect of DON (*p* < 0.05) on day 1 compared to the control and the FB1 groups. However, there was no gene activation in the FB1-treated group (Table 7). The gene expression of *GSS* was significantly lower on the first day in DON (*p* < 0.05) and FB1 (*p* < 0.05) groups than in the control group, but it was higher on day 2 in the FB1-treated group (*p* < 0.05) compared to the control and the two trichothecene-treated groups. On day 3, higher relative gene expression of *GSS* was found in the T-2 toxin-treated group (*p* < 0.05) compared to the other experimental groups (Table 7). There were no significant changes in the gene expression of *GSR* (Table 7).

## 4. Discussion

The presence of mycotoxins that contaminate feed is one of the primary problems in the production of animal products; that is why it is particularly important to know their mechanism of action in the animal body. Three *Fusarium* mycotoxins, T-2 toxin, DON, and FB1, have a well-known effect on the activation of oxidative stress and lipid peroxidation, which the glutathione redox system can neutralize.

According to the results, none of the examined mycotoxins initiated measurable lipid peroxidation; possibly, the applied dose did not cause even mild oxidative stress in the liver of laying hens. On the contrary, a significant decrease in lipid peroxidation parameters, such as the CD levels, as an effect of all three examined mycotoxins and a significant decrease in MDA concentration as an effect of DON were observed. These decreases may be due to a change in the fatty acid composition of liver tissue lipids as the effect of the mycotoxin exposure, which has been detected previously as the effect of FB1 and T-2 toxins in rabbits [45]. The altered fatty acid composition can affect the sensitivity of lipids to oxidation by oxygen free radicals [46]. Markers of the glutathione redox system, amount of GSH, and activity of GPx were also decreased compared to the control at the end of the trial as an effect of FB1, but the T-2 toxin and DON did not have the same effect. This difference may be due to the rapid activation of the glutathione redox system as the effect of FB1, which effectively inhibited lipid peroxidation but resulted in a rapid depletion of the glutathione redox system. It seems that there was not enough time available for the regeneration of glutathione disulfide and de novo synthesis and/or post-translational activation of GPx. This result is supported by a previous study [47], where FB1 was found to decrease the GSH concentration in the liver because of the removal of free radicals as caused by oxidative stress. However, DON or T-2 toxin at the dose applied did not activate the glutathione system, possibly due to lack of even mild oxidative stress.

As a result of the mycotoxin exposure, significant differences were found in the relative expression of genes involved in regulating the glutathione redox system: induction for *GPX4* in the group treated with T-2 toxin and DON after 24 h of mycotoxin exposure but not in the FB1-treated group. This result suggested that the trichothecene mycotoxins, T-2 toxin, and DON rapidly activated the antioxidant defense system at the gene expression level, preventing further lipid peroxidation processes even if the given mycotoxins otherwise induce the formation of reactive oxygen species (ROS). On the other hand, FB1 did not activate the glutathione redox system at gene expression level in all cases, suggesting tolerance for the applied dose of FB1 in laying hens. However, the relative expression of the *GSS* gene increased in the liver of laying hens on day 2 in the FB1-treated group and on day 3 as an effect of T-2 toxin. According to the differences in the changes of relative gene expression, T-2 toxin and DON at the dose applied induce a rapid ROS formation and then a rapid and effective antioxidant response. At the same time, FB1 induces less ROS formation; therefore, the relative expression of not all of the glutathione redox system encoding genes increased. These results suggest that short-term mycotoxin exposure reached the threshold for activation of the expression of some, but not all, of the antioxidant genes. Still, it was detectable only at the mRNA level, not at the activity level, during the 3-day trial.

These results can be helpful for multi-mycotoxin examinations, as these mycotoxins frequently co-occur in feedstuffs, and combined exposure might cause additive, synergistic, or antagonistic toxic reactions. Short-term effects of T-2 toxin, DON, and FB1 in poultry are not fully described yet, and their effects on ROS formation are contradictory. The burning question is still open: how long of a period is required after DON, T-2 toxin, and FB1 uptake for the significant changes at the cellular level to elevate oxidative stress? Further studies are required to investigate the order of the response of biochemical and molecular markers of oxidative stress in the case of multi-mycotoxin exposure.

## 5. Conclusions

In conclusion, short-term and relatively low-dose T-2 toxin, DON, and FB1 exposure reduced lipid peroxidation by the 72nd hour of the experiment. The results show that the mycotoxins used might have influenced the fatty acid composition of the membrane phospholipids and thus their susceptibility to oxidation. Furthermore, the examined mycotoxins affected the relative gene expression of the genes encoding the enzymes of the glutathione redox system, but this effect did not manifest at the level of GSH amount and GPx activity. Thus, the results suggest that the doses applied did not cause oxidative stress in laying hens or were effectively eliminated by the glutathione redox system.

## Figures and Tables

**Table 1:** Predicted and measured mycotoxin concentrations in the diets (mg/kg feed).

Group	Predicted	Measured
Control	-	<0.02
T-2/HT-2 toxin	0.5	0.43/0.19
DON/3-AcDON/15-AcDON	10	10.68/-/-
FB1	40	39.73

**Table 2:** Calculated nutrient content of the laying hen diet (%).

Nutrient	Content
Dry matter	89.20
Crude protein	16.10
Ether extract	2.50
Crude fiber	5.50
Lysine	0.79
Methionine	0.38
Methionine + Cysteine	0.71
Calcium	4.12
Phosphorus (available)	0.48
Sodium	0.17
ME (MJ/kg)	11.97

**Table 3:** Primers of target and endogenous genes.

Real-Time PCR Primers of Target and Endogenous Genes			
Gene	Forward (5′–3′)	Reverse (5′–3′)	GenBank Accession No.
*GAPDH*	TGACCTGCCGTCTGGAGAAA	TGTGTATCCTAGGATGCCCTTCAG	NM_204305.1
*GPX4*	AGTGCCATCAAGTGGAACTTCAC	TTCAAGGCAGGCCGTCAT	NM_001346448.1
*GSS*	GTACTCACTGGATGTGGGTGAAGA	CGGCTCGATCTTGTCCATCAG	XM_425692.6
*GSR*	CCACCAGAAAGGGGATCTACG	ACAGAGATGGCTTCATCTTCAGTG	XM_015276627.2

**Table 4:** MGB-NFQ dual-labeled probes of target and endogenous genes.

Gene	MGM Dual Labelled Probe	Fluorescent Dye
*GAPDH*	CCAGCCAAGTATGATGAT	VIC
*GPX4*	CAGCCCAATGGAG	FAM
*GSS*	AGGAGGGAACAACCTG	FAM
*GSR*	CTGGCACTTCGGCTC	FAM

**Table 5:** Short-term effect of T-2 toxin, deoxynivalenol (DON), and fumonisin B_1_ (FB1) treatment on the parameters of lipid peroxidation in liver of laying hens (mean ± S.D.; *n* = 6).

**Conjugated Dienes (OD 232 nm)**				
	**Day 0**	**Day 1**	**Day 2**	**Day 3**
Control	0.31 ± 0.03	0.29 ± 0.02	0.29 ± 0.01	0.32 ^b^ ± 0.03
T-2 toxin		0.28 ± 0.02	0.28 ± 0.02	0.27 ^a^ ± 0.01
DON		0.29 ± 0.02	0.30 ± 0.01	0.27 ^a^ ± 0.02
FB1		0.29 ± 0.03	0.29 ± 0.04	0.27 ^a^ ± 0.02
**Conjugated Trienes (OD 268 nm)**				
	**Day 0**	**Day 1**	**Day 2**	**Day 3**
Control	0.16 ± 0.01	0.15 ± 0.01	0.14 ± 0.01	0.15 ± 0.01
T-2 toxin		0.14 ± 0.01	0.15 ± 0.01	0.13 ± 0.01
DON		0.15 ± 0.01	0.15 ± 0.01	0.14 ± 0.01
FB1		0.15 ± 0.01	0.15 ± 0.03	0.14 ± 0.01
**Malondialdehyde (μmol/g Wet Weight)**				
	**Day 0**	**Day 1**	**Day 2**	**Day 3**
Control	54.12 ± 11.02	47.36 ± 9.2	38.76 ± 8.46	41.83 ^b^ ± 8.78
T-2 toxin		52.69 ± 13.11	42.87 ± 12.29	40.64 ^b^ ± 9.71
DON		50.91 ± 11.16	42.50 ± 9.25	27.71 ^a^ ± 5.63
FB1		44.04 ± 10.46	39.05 ± 7.72	37.17 ^ab^ ± 4.77

^a,b^ Means with different letters in the same column differ significantly (*p* < 0.05).

**Table 6:** Short-term effect of T-2 toxin, deoxynivalenol (DON), and fumonisin B_1_ (FB1) treatment on the parameters of glutathione redox system in liver of laying hens (mean ± S.D.; *n* = 6).

**Reduced Glutathione (μmol/g Protein)**				
	**Day 0**	**Day 1**	**Day 2**	**Day 3**
Control	4.43 ± 0.66	5.26 ± 0.66	5.89 ± 1.18	7.89 ^b^ ± 1.19
T-2 toxin		5.83 ± 0.79	6.06 ± 0.97	8.02 ^b^ ± 0.68
DON		5.40 ± 0.46	6.28 ± 1.41	8.21 ^b^ ± 0.91
FB1		5.30 ± 0.93	6.55 ± 0.78	5.67 ^a^ ± 1.11
**Glutathione Peroxidase (U/g Protein)**				
	**Day 0**	**Day 1**	**Day 2**	**Day 3**
Control	4.43 ± 0.44	5.41 ± 0.64	6.17 ± 0.66	8.04 ^b^ ± 1.19
T-2 toxin		5.37 ± 0.48	5.88 ± 0.93	7.72 ^b^ ± 0.79
DON		5.38 ± 0.48	6.39 ± 0.99	8.57 ^b^ ± 0.94
FB1		5.65 ± 0.45	6.46 ± 0.86	5.67 ^a^ ± 1.29

^a,b^ Means with different letters in the same column differ significantly (*p* < 0.05).

**Table 7:** Short-term effect of T-2 toxin, deoxynivalenol (DON), and fumonisin B_1_ (FB1) treatment on the relative gene expression of the members of the glutathione redox system in the liver of laying hens (mean ± S.D.; *n* = 6; equal amounts of cDNA per animal).

**Glutathione Peroxidase 4 (*GPX4*)**				
	**Day 0**	**Day 1**	**Day 2**	**Day 3**
Control	1.00 ± 0.03	0.98 ^a^ ± 0.09	1.03 ± 0.04	1.21 ^a^ ± 0.12
T-2 toxin		1.46 ^b^ ± 0.09	1.04 ± 0.22	1.48 ^b^ ± 0.07
DON		1.34 ^b^ ± 0.12	1.27 ± 0.07	1.40 ^ab^ ± 0.08
FB1		1.01 ^a^ ± 0.11	1.16 ± 0.10	1.19 ^a^ ± 0.13
**Glutathione Synthetase (*GSS*)**				
	**Day 0**	**Day 1**	**Day 2**	**Day 3**
Control	1.01 ± 0.16	1.37 ^b^ ± 0.19	0.87 ^a^ ± 0.18	0.95 ^ab^ ± 0.24
T-2 toxin		1.08 ^ab^ ± 0.16	0.83 ^a^ ± 0.13	1.19 ^b^ ± 0.14
DON		0.75 ^a^ ± 0.15	1.00 ^a^ ± 0.14	0.60 ^a^ ± 0.16
FB1		0.82 ^a^ ± 0.13	1.36 ^b^ ± 0.19	0.81 ^b^ ± 0.11
**Glutathione Reductase (*GSR*)**				
	**Day 0**	**Day 1**	**Day 2**	**Day 3**
Control	1.07 ± 0.45	1.33 ± 0.28	0.87 ± 0.21	1.06 ± 0.63
T-2 toxin		1.04 ± 0.29	1.01 ± 0.38	1.15 ± 0.84
DON		1.31 ± 0.53	0.98 ± 0.19	1.17 ± 0.40
FB1		0.96 ± 0.38	1.11 ± 0.61	0.96 ± 0.14

^a,b^ Means with different letters in the same column differ significantly (*p* < 0.05).

## Data Availability

Data is contained within the article.
